# Non-sterilizing, Infection-Permissive Vaccination With Inactivated Influenza Virus Vaccine Reshapes Subsequent Virus Infection-Induced Protective Heterosubtypic Immunity From Cellular to Humoral Cross-Reactive Immune Responses

**DOI:** 10.3389/fimmu.2020.01166

**Published:** 2020-06-09

**Authors:** Angela Choi, Lorena I. Ibañez, Shirin Strohmeier, Florian Krammer, Adolfo García-Sastre, Michael Schotsaert

**Affiliations:** ^1^Department of Microbiology, Icahn School of Medicine at Mount Sinai, New York, NY, United States; ^2^Graduate School of Biomedical Sciences, Icahn School of Medicine at Mount Sinai, New York, NY, United States; ^3^Instituto de Ciencia y Tecnología Dr. César Milstein, CONICET, Ciudad de Buenos Aires, Buenos Aires, Argentina; ^4^Global Health and Emerging Pathogens Institute, Icahn School of Medicine at Mount Sinai, New York, NY, United States; ^5^Division of Infectious Diseases, Department of Medicine, Icahn School of Medicine at Mount Sinai, New York, NY, United States; ^6^The Tisch Cancer Institute, Icahn School of Medicine at Mount Sinai, New York, NY, United States

**Keywords:** influenza, pre-existing immunity, TIV, alveolar macrophage, tissue-resident memory T cell, germinal center B cell, heterosubtypic immunity

## Abstract

Conventional influenza vaccines aim at the induction of virus-neutralizing antibodies that provide with sterilizing immunity. However, influenza vaccination often confers protection from disease but not from infection. The impact of infection-permissive vaccination on the immune response elicited by subsequent influenza virus infection is not well-understood. Here, we investigated to what extent infection-permissive immunity, in contrast to virus-neutralizing immunity, provided by a trivalent inactivated virus vaccine (TIV) modulates disease and virus-induced host immune responses after sublethal vaccine-matching H1N1 infection in a mouse model. More than one TIV vaccination was needed to induce a serum HI titer and provide sterilizing immunity upon homologous virus infection. However, single TIV administration provided infection-permissive immunity, characterized by lower viral lung titers and faster recovery. Despite the presence of replicating virus, single TIV vaccination prevented induction of pro-inflammatory cyto- and chemokines, alveolar macrophage depletion as well as the establishment of lung-resident B and T cells after infection. To investigate virus infection-induced cross-protective heterosubtypic immune responses in vaccinated and unvaccinated animals, mice were re-infected with a lethal dose of H3N2 virus 4 weeks after H1N1 infection. Single TIV vaccination did not prevent H1N1 virus infection-induced heterosubtypic cross-protection, but shifted the mechanism of cross-protection from the cellular to the humoral branch of the immune system. These results suggest that suboptimal vaccination with conventional influenza vaccines may still positively modulate disease outcome after influenza virus infection, while promoting humoral heterosubtypic immunity after virus infection.

## Introduction

Over the course of a lifetime, humans are repeatedly exposed to influenza virus by natural infection or vaccination. Due to its error prone replication complex, influenza virus acquires mutations (antigenic drift) that allow it to evade pre-existing host immune responses. As a result, the virus is responsible for causing annually recurrent respiratory disease worldwide (1). Additionally, influenza virus can exchange gene segments (antigenic shift), generating each 10–50 years novel pandemic influenza viruses that the human population is naïve to. Vaccines are the best method of protection, but are strain specific. Thus, annual re-formulation and re-administration of the vaccine is necessary. The repeated infection and vaccination people undergo throughout life leads to the buildup of influenza-specific immunity in individuals.

There are different immune mechanisms that play a role in providing protection against influenza virus. Most notable are neutralizing antibodies. Influenza virus vaccines are formulated to induce neutralizing antibodies toward the surface glycoprotein, hemagglutinin (HA). Measurement of influenza-specific neutralizing antibodies through hemagglutination inhibition (HAI) assay is used as the gold standard correlate of protection (2). However, during seasons with antigenic mismatch, influenza virus can escape previously induced HA-specific neutralizing antibodies. In the absence of neutralizing antibodies, there are other immune mechanisms that contribute to protection against influenza-related disease. Cytotoxic T lymphocytes, non-neutralizing antibodies, and innate immune responses are few examples that also play a role in providing immunity. Although protective from disease, these immune responses are often infection-permissive. Because they do not fully neutralize the virus, viral replication still occurs and virus-host interactions can be initiated. Protection provided by these immune mechanisms is often hard to predict as they rely on multiple mechanisms that act synergistically and adequate assays to measure how they correlate with protection are not always available. Because of these reasons, measuring serum antibodies with the potential of virus neutralization by HAI assay does not always accurately represent the protection status of an individual and other correlates of protection should be considered.

It is known that pre-existing immunity toward influenza virus contributes to protection during re-infection in later seasons with an influenza virus of a different subtype (heterosubtypic immunity, HSI). Repeated exposure to influenza virus in humans has been shown to correlate with protection from severe disease during re-exposure to a different subtype of influenza virus (3). Both virus-induced humoral and cellular adaptive immune responses can contribute to such heterosubtypic immunity (4–8). Repeated vaccinations against influenza virus can also modulate both humoral and cellular immune responses in individuals (9, 10). Overall, the degree and type of pre-existing immunity can affect host immune responses after influenza virus infection. Many efforts are currently ongoing to investigate immune mechanisms that can provide long-lasting broad protection against influenza viruses. This is important for rational approaches to broaden and prolong the protective scope of current influenza virus vaccines and for the development of universal influenza virus vaccines (11–13). Many preclinical studies have investigated the correlation between long term protection and virus-induced host immune responses, which include induction of cross-reactive antibodies, presence of tissue resident memory T cells (TRM) and formation of tertiary lymphoid organs in the respiratory tract (14–17). Most experimental models, however, use naïve animals to deliver proof of concept. Studying virus- or vaccine-host immune responses in the presence of pre-existing immunity toward influenza virus would be more relevant to the human situation. In this work, we investigated the impact of pre-existing immunity, provided by intramuscular vaccination or transfer of immune serum from vaccinated mice, on host immune responses toward influenza virus infections. We show that such pre-existing immunity skews host-immune responses upon influenza H1N1 infection and changes correlates of heterosubtypic immunity during re-infection with H3N2 influenza virus. These findings provide additional information for the development of influenza vaccination strategies with the potential to provide broad protection against influenza viruses of different subtypes.

## Materials and Methods

### Study Design (Mouse Vaccination and Challenge Scheme)

Female, 6–8 weeks old BALB/c mice were obtained from Charles River Laboratories, Wilmington, MA, and housed under specified pathogen free conditions with food and water ad libitium. All experiments were approved by and performed according to the guidelines of Icahn School of Medicine at Mount Sinai Institutional Animal Care and Use Committee (IACUC-2017-0330).

For one group, mice were vaccinated with a seasonal trivalent inactivated influenza virus vaccine (TIV) (Fluzone 2005 – 2006 formula, Aventis) adjuvanted with Alhydrogel (InvivoGen) a total of three times. The vaccine used in this study was 2005–2006 formula that contains a H1N1 component, H3N2 component, and a B component [A/New Caledonia/20/1999/IVR-116 (H1N1), A/New York/55/2004/X-157 (H3N2) [an A/California/7/2004 (H3N2)-like strain] and B/Jiangsu/10/2003 (a B/Shanghai/361/2002-like strain)].

TIV and adjuvant mixture was given intramuscularly to both hind legs (50 ul/leg; 100 ul/mouse) each time with an interval of 3 weeks in between (Day−63,−42, and−21). The other two groups of mice were only vaccinated once with the same amount of either TIV or 1X PBS, both also adjuvanted with Alhydrogel (Day -21).

Three weeks later, all groups were challenged (Day 0). The group which received three vaccinations (3X TIV) was challenged with mouse-adapted A/New Caledonia/20/1999 H1N1 (NC99). Mice that received only one vaccination were further split into two groups where one group received a sublethal dose (0.2LD_50_) of H1N1 NC99 virus infection and the other received a mock infection of egg allantoic fluid. Four weeks later (Day 28), all five groups (3X TIV NC, TIV Mock, TIV NC99, PBS Mock, and PBS NC99) received a lethal dose (5 LD_50_) of H3N2 reassortant virus, which contains the HA and NA molecules from A/HK/8/68 H3N2 virus and the internal gene products of the PR8 backbone (X-31). For each challenge, mice were anesthetized with a mild ketamine and xylazine mixture and either virus or egg allantoic fluid (50 ul/mouse) was given intranasally. Body weight loss after infection was recorded as a read-out for morbidity.

### ELISA and HAI Assay

Sera from mice were collected 14 days after final vaccination (day 7) and 26 days after first challenge with H1N1 NC. To get serum from each mouse, mice were bled submandibular and the collected blood was left at 4°C overnight. Then the samples were centrifuged twice at 16,000 rcf for 15 min, collecting serum after each spin.

ELISAs were performed using 96-well-microtiter plates (ThermoFisher Scientific) coated overnight at 4°C with 50 ul of in house produced baculovirus-expressed recombinant trimeric HA or NP protein (diluted in carbonate/bicarbonate buffer) at a concentration of 2 ug/ml. After plates were washed with phosphate buffered saline with 0.1% Tween-20 (PBS-T), plates were blocked with blocking buffer (PBS with 1% BSA) for 1 h at room temperature. Serum samples diluted to 1:100 in sample buffer (PBS-T + 0.5% BSA) were added 50 ul per well to the plates after blocking buffer was removed. Plates were incubated at room temperature for 2 h. Later, plates were washed again with PBS-T and 50 ul of secondary antibody, horseradish peroxidase-conjugated sheep-derived anti-mouse IgG (GE Healthcare) diluted in PBS-T were incubated at room temperature for 1 h. Plates were washed again when incubation was done and developed with 50 ul 3, 3′, 5, 5′-Tetramethylbenzidine (TMB) per well. Once blue color is seen, the development was stopped by adding 50 ul of 1M H_2_SO_4_. Plates were read at 450 nm and data analyses were done using Graphpad Prism software.

For hemagglutinin inhibition assay (HAI), individual serum samples from each group were inactivated by receptor destroying enzyme (RDE) (Denka Seiken Co., LTD.) treatment overnight at 37°C. Afterwards, sera were treated with 2.5% sodium citrate for an hour at 56°C and 1X PBS was added to make the final serum dilution to be 1:10. Following the WHO protocol, serum samples were 2-fold serial diluted in 1X PBS in a V-bottom 96-well-plate. Virus titer of 4HAU/25 ul in 1X PBS was added to each well of the plate. Virus and sera were incubated for 15 min at room temperature before adding 0.5% chicken red blood cells (Lampire Biological Laboratories). Plates were incubated at room temperature for 30 min and HAI results were recorded.

### Western Blot

To probe for NP content in the seasonal TIV (Fluzone), proteins from the vaccine were separated on a 4–12% gradient polyacrylamide gel (Bio Rad) and transferred to a PVDF membrane (Milipore). The membrane was then blocked in 5% milk in PBS for 1 h at room temperature. Immunodetection was performed using anti-NP (Millipore) antibody (1:1000 dilution) followed by secondary anti-mouse IgG antibody conjugated to horseradish peroxidase (GE Healthcare) (1:5000 dilution). NP specific band was detected by ECL western blotting detection system (ThermoFisher Scientific).

### IFN Alpha and Beta ELISA

The level of interferon (IFN) alpha and beta in lungs were measured using Pbl Assay Science Verikine Mouse IFN alpha ELISA kit and IFN beta ELISA Kit. Briefly, 100 ul of lung homogenates were added to the pre-coated IFN-alpha microtiter plates as well as 50 ul of antibody solution. The plate was then incubated overnight at 4°C. After incubation, the plate was washed 3 times with diluted wash solution before adding 100 ul of horse-radish peroxidase (HRP) solution. The plates were washed again after 2 h of incubation at room temperature. One hundred microliter of TMB substrate was added to the plate and incubated in the dark at room temperature to develop for 15 min. The development was stopped by adding 100 ul stop solution to each well. Using a microplate reader, the plate was read at absorbance 450 nm. The protocol for the IFN beta ELISA was similar as above. But after 100 ul lung homogenates were added to each well the plates were incubated at 4°C overnight. Then 100 ul of antibody solution was added and incubated at room temperature for 1 h followed by another hour of incubation of 100 ul of HRP solution with washing the plate three times with wash solution in between each step. The development and reading of the IFN-beta ELISA plate was the same as the IFN-alpha ELISA plate.

### Cytokine/Chemokine Measurements

Supernatant from lung homogenates collected from mice were used for the assay. Cytokine and chemokine measurements were done through ProcartaPlexTMA multiplex assay (Invitrogen). Twenty-five ul of undiluted sample were added to each well of the flat bottom 96-well-plate along with the same amount of magnetic capture beads. The plate was incubated overnight at 4°C. Afterwards, the plate was washed 3 times with wash buffer using a magnetic plate washer and incubated with 12.5 ul of diluted detection antibody for 30 min at room temperature. Plates were washed again before adding 12.5 ul of streptavidin-PE solution. After incubating for another 30 min at room temperature, the plate was washed again and beads were resuspended in 120 ul or reading buffer. Data were acquired using the MagpixTM.

### Viral Lung Plaque Assay

To quantify viral lung titers after challenge, mice were euthanized and lungs were harvested. The lungs were collected directly in cryotubes on dry ice and were stored at −80°C. The day of the assay, lungs were homogenized in 0.6 ml 1X PBS. After homogenization, lungs were centrifuged at max speed at 4°C for 15 min. The supernatants of each sample were collected separately and aliquoted. Titers of infectious particles were determined by plaque assay. In short, lung homogenates were 10-fold serial diluted in 1X PBS. In a 12-well-tissue culture plate (name), 250 ul of each dilution was used in each well to infect Madin-Darby Canine Kidney (MDCK) cells for 1 h at 37°C. After, cells were washed once with 1XPBS and overlayed with a mixture of 2% Oxoid agar (Oxoid Ltd.) and sodium bicarbonate buffered serum free 2X minimum essential medium (MEM) supplemented with 1% diethylaminoethyl (DEAE)-dextran and tosylamide-2-phenylethyl chloromethyl ketone (TPCK)-treated trypsin. Plaque assay plates were incubated at 37°C for 48 h and were immunostained with polyclonal serum and secondary horseradish peroxidase-conjugated sheep-derived anti-mouse IgG (GE Healthcare). Final viral titers were determined after plates were developed with TrueBlue substrate (KPL-Seracare).

### Evans Blue Staining

To observe lung integrity and edema after primary challenge, Evans Blue (Sigma) was given to 3 mice per group (except the 3xTIV NC99 group) 6 days after first infection. After mice were knocked down, mice received 100 μl of Evans Blue (2% w/v in PBS) through retro-orbital injections and were given few minutes for the dye to circulate in the body. Bronchoalveolar lavage fluid (BALF) of about 500 ul was collected from each mouse and 100 ul of BALF was plated on a microtiter plate. The plate was read at absorbance of 610 nm and data were plotted on Graphpad Prism software.

### ELISpot

Lungs from mice were forced through 70 um cell strainer. The erythrocytes in the lung cell suspension were then lysed using red blood cell (RBC) lysis buffer. The cell pellets were resuspended in 33% Percoll PLUS (GE Healthcare) and centrifuged at 400 × g for 5 min. Carefully, the supernatant was removed and the pelleted cells enriched for lymphocytes were resuspended in 450 ul full RPMI media [10% FBS, 1% p/s, RPMI media (gibco)]. Pre-coated anti-mouse IFN-γ microplates (R&D systems) were blocked with full RPMI media for 20 min before 40 ul of lung cell suspension were plated in each well. Lymphocytes from each group were restimulated with 10-fold serial dilutions starting from 1 to 10–6 ug/well of MHCI-binding H2d-restricted NP-derived TYQRTRALV peptide (NP155) (Think Peptides) and 1 ug/well of an irrelevant RSV F protein H-2K(d)-restricted epitope KYKNAVTEL peptide as a negative control. The restimulated microplate was incubated overnight at 37°C. The following day, the microplate was washed 3 times in provided wash buffer after the cells were removed. Then 100 ul of diluted detection antibody were added to each well and incubated at room temperature for 2 h. The plates were washed again 3 times with wash buffer and 100 ul of diluted Streptavidin-AP were added to each well. After the plate was incubated for 1 h at room temperature, it was washed again 3 times before adding 100 ul of chromogenic substrate into each well. Spots were seen after 40 min of development. Thus, substrate was removed from the plate and washed with distilled water. After the plate was completely dry, images of the each well were taken by CTL ELIspot reader. Quantification of spots was done using ImageJ.

### Flow Cytometry

After samples were collected and stained with specific antibodies listed below, flow cytometric data were acquired with a Gallios (Beckman Coulter) flow cytometer with Kalluza software. Data analysis was further performed using FlowJo X.0.7 (Treestar) software and the R language and environment for statistical computing, R Development Core Team, 2009 (R Foundation for Statistical Computing, Vienna, Austria (ISBN 3-900051-07-0, URL http://www.R-project.org). Exported flow cytometry data from FlowJo were further analyzed in R using the R/Bioconductor packages flowCore and matrixStats. Marker intensities were arcsinh-transformed (cofactor = 150) and scaled between values 0 to 1 for visualization. Packages ConsensusClusterPlus and FlowSOM are used for cell clustering and visualized using the packages Rtsne, ggplot2, pheatmap, and RcolorBrewer. Quantitative data were visualized using Graphpad Prism version 7.00 for Windows (GraphPad Software, San Diego, CA, USA).

#### Myeloid Cell Populations in Lungs

On day 7, lungs were collected aseptically after mice were euthanized. Lungs were passed through 70 um cell strainer to obtain single-cell homogenates. Red blood cells were lysed and the remaining cells were washed with FACS buffer. After cells were resuspended in 200 ul of FACS buffer, Fc receptors were blocked with anti-CD16/CD32 (Fc Block, BD) and then stained using the following antibodies (all from eBiosciences): anti-CD11b-APC, anti-CD11c-PECy7, anti-CD45-AF700, anti-Gr1-PerCpCy5.5, viability-e520, anti-Ly6C-PE, anti-MHCII-e450, anti-SiglecF-PE-CF594.

#### NP-Specific CD8+ T Cells in Lungs

Mice lung lymphocytes collected for the ELISPOT assay (R&D Systems) were also used to stain NP-specific CD8+ T cells. After lymphocytes were pelleted and resuspended in FACS buffer, Fc receptors were blocked using anti-CD16/CD32 (BD) and were stained using viability stain-e520, anti-MHCII-e450, anti-CD3-APC-Cy7, anti-CD8-APC (all eBiosciences), and NP-specific Pro5 pentamer-PE (NP155-peptide, ProImmune).

#### Germinal Center B Cell in Lungs and Mediastinal Lymph Nodes

To look at germinal center formation in mice, lungs and lymph nodes were harvested 24 days after the first challenge with NC99 H1N1. Lungs from each mouse were processed through 70 um cell strainer separately, red blood cells were lysed, and resuspended in FACS buffer. Collected lymph nodes from mice were pooled per group and made into single-cell homogenates by forcing them through a cell strainer. To stain the cells, Fc receptor were blocked using anti-CD16/CD32 (BD), anti-B220-AF700, anti-GL7-e420, anti-Fas-PE Cy7, anti-IgM-PerCpCy5.5, and anti-CD3-APC (all eBioscience) were used.

#### Lung Resident CD8+ T Cells

To look at virus induced lung resident memory CD8+ T cell response, lungs at 27 days after the initial challenge were analyzed from each group (*n* = 5 mice/group but *n* = 4 mice/ 3X TIV NC). Before the lungs were harvested, anti-CD45 antibody (AF700 from eBioscience; 3 μg/mouse in 100 μl PBS) were given retro-orbitally after mice were knocked down with pentobarbital. Immediately after, lungs were harvested and single-cell suspension in 1X PBS were made by forcing lungs through 70 um cell strainer. After lung cell suspensions were treated with red blood cell lysis buffer, they were stained with anti-CD44-PECy7, anti-CD3-FITC, anti-CD8-PerCP, anti-CD103-APC, anti-CD69-PE-CF594, and viability dye-e450 (all eBioscience) along with Fc receptor blocking anti-CD16/CD32 (BD).

### 3x TIV Vaccination T Cell Study Design

Groups of mice were either vaccinated three times with TIV or 1X PBS. Vaccinations were given at 3 week intervals, intramuscularly to both hind legs. Twenty one days after the last vaccination, each vaccination groups were further divided by challenging them with a sublethal dose (0.2 LD_50_) of NC H1N1 or egg allantoic fluid. Lungs and spleens were collected and prepared into single-cell suspensions. T cell responses were monitored by flow cytometry and ELISPOT assay as described above.

### Passive Transfer Challenge Experiment

Two groups of 25 6–8weeks old female BALB/c mice received two vaccinations 2 weeks apart. They were given either 50 ul TIV or 1X PBS (mock) intramuscularly in both hind legs (total 100 ul, 3 ug each HA) each vaccination. Terminal bleeds were performed 14 days after the boost to collect serum. For the passive serum transfer and challenge study, the collected sera from twice TIV or mock vaccinated mice were pooled separately. Then 200 ul of pooled serum were passively transferred intraperitoneally (*n* = 5 mice per group). One day after the serum transfer, both groups were challenged intranasally with 0.2 LD_50_ H1N1 NC99. Ten days after the infection, mice were euthanized and lungs were harvested for IFN-y ELISpot analysis (R&D Systems).

### *In vitro-in vivo* Neutralization Assay

Sera were collected from each group 26 days after their first challenge with either NC99 or egg allantoic fluid (Mock). Serum samples were pooled by group and incubated with the same lethal dose of X31 H3N2 virus (2000 PFU) for 1 h at 37°C. The combination of serum-virus mixture was then given intranasally to naïve mice. Morbidity and mortality were monitored for 14 days. 3X TIV NC *n* = 3, TIV Mock *n* = 3, TIV NC *n* = 5, PBS Mock *n* = 4, PBS NC *n* = 5.

### Statistics

All statistical analyses were performed using Graphpad Prism version 7.00 for Windows (GraphPad Software, San Diego, CA, USA) and with the R language and environment for statistical computing, R Development Core Team, 2009 (R Foundation for Statistical Computing, Vienna, Austria (ISBN 3-900051-07-0, URL http://www.R-project.org). Statistical significance levels for ELISA data were computed by one-way ANOVA tests followed by a Tukey post test. Statistical significance levels for all other assays were calculated by non-parametric Kruskall-Wallis tests followed by a Dunn's post test. Comparisons of multiple groups were performed against control groups (PBS-Alum after vaccination and PBS NC99 after infection). Significance levels are indicated with asterisks: ^*^p < 0.05, ^**^p < 0.01, ^***^p < 0.001, and ^****^p < 0.0001.

## Results

**Figure 1:**
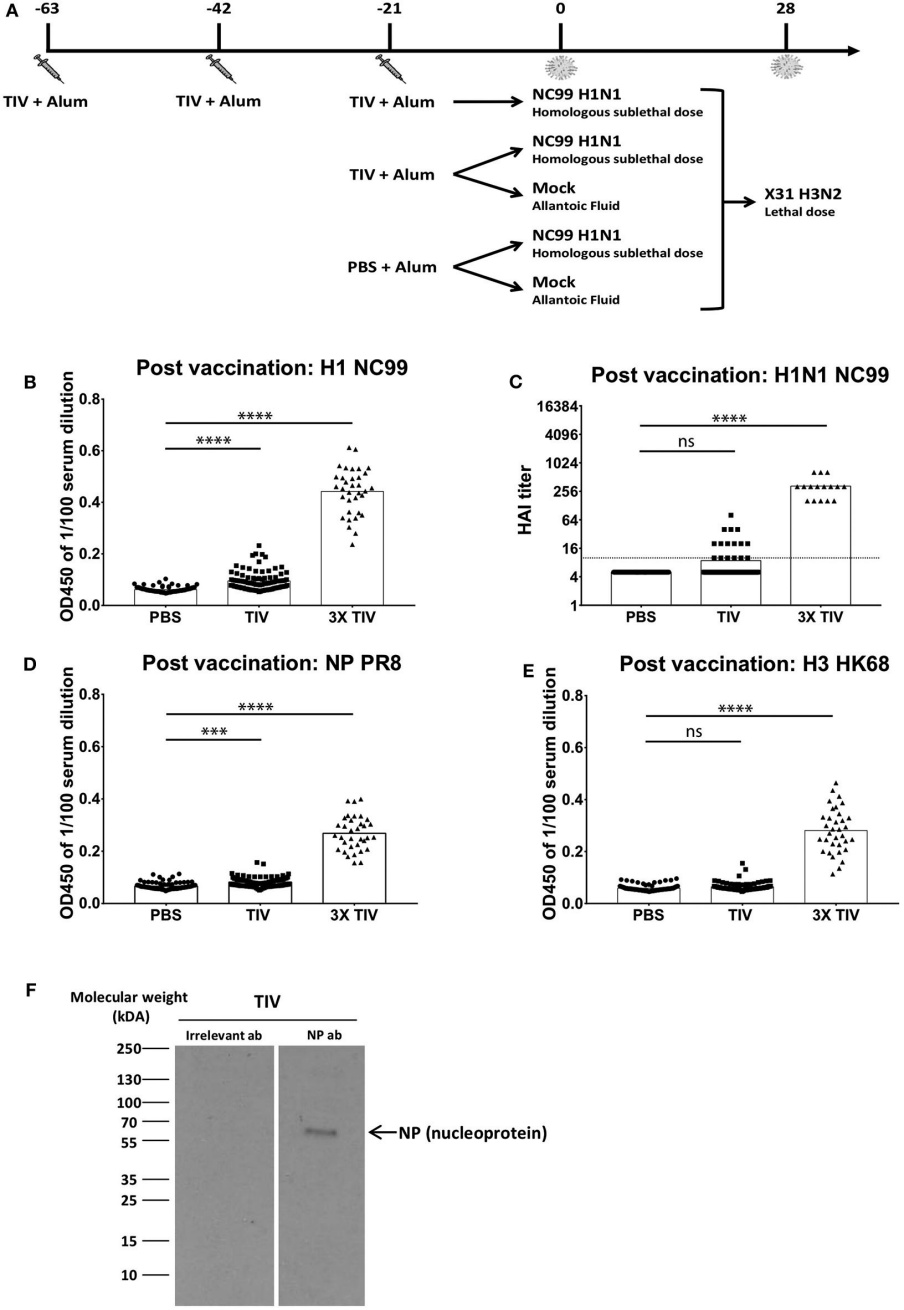
More than one TIV vaccination is needed to induce virus-specific antibody responses efficiently in naïve mice. **(A)** Overall study design. BALB/c mice were either vaccinated three times with seasonal TIV, once with TIV, or once with PBS. Each vaccination was adjuvanted with Alum. Three weeks after the last immunization, animals were either challenged with H1N1 NC99 virus or allantoic fluid (mock challenge). Four weeks after the initial challenge, animals were challenged a second time with a lethal dose of X-31 H3N2. **(B,D,E)** Individual mouse sera were collected 14 days after the last vaccination. Samples were diluted 1/100 for ELISA. Total IgG antibody levels were measured against recombinant full length HAs (H1 NC99 and H3 HK68) and NP PR8. Each symbol represents a single mouse. Bars represent means. **(C)** Antibodies post vaccination sera were tested against H1N1 NC99 virus for HAI titers. Dotted line represents the limit of detection. Each symbol represents one mouse. Bars represent means. **(F)** Using NP antibody, presence of NP in TIV was confirmed through western blot. ***p < 0.001, ****p < 0.0001, ns = not significant.

An outline of the vaccination/infection experiments conducted in our studies is given in Figure 1A. Mice were vaccinated with an alum adjuvanted seasonal trivalent virus vaccine (TIV) via the intramuscular route. The A/New Caledonia/20/1999 H1N1 virus strain was used for the influenza vaccine formulation during the influenza seasons of 2000 until 2006. Therefore, individuals who were annually vaccinated during those seasons have been exposed to the same vaccine antigen more than once. Thus, to mimic repeated vaccination, a group of mice received TIV three times (3x TIV) with three weeks interval, while another group received a single dose of TIV. As a control, a third group was vaccinated with phosphate buffered saline (PBS). Three weeks after the last vaccination, mice were either challenged with a sublethal dose of A/New Caledonia/20/1999 (abbreviated as NC99) H1N1 virus that matches the vaccine strain or mock challenged. After NC99 challenge, mice were allowed to recover for 4 weeks before being rechallenged with a lethal dose of X-31 H3N2 virus, which has antigenically different HA and NA surface proteins from the H3N2 component of the TIV (influenza A/New York/55/2004/X-157 virus). Host immune responses to vaccination and infection were monitored at different time points as mentioned in the text.

### More Than One TIV Vaccination Is Needed to Efficiently Induce Virus-Specific Antibody Responses in naïve Mice

Seasonal TIV is formulated to induce influenza virus HA-specific antibodies in humans. To assess if antibodies were induced in mice after vaccination, sera were collected at 2 weeks post last vaccination and diluted at 1/100 to perform enzyme-linked immunosorbent assay (ELISA). Total IgG titers against recombinant NC99 H1 hemagglutinin (HA), were absent in the control group that received PBS instead of TIV. Mice that received three TIV vaccinations (3x TIV) showed the highest titer. Mice that received TIV once, however, did not always seroconvert against H1 (Figure 1B). This is also reflected in the hemagglutination inhibition (HAI) titer against the NC99 H1N1 virus (Figure 1C). All mice that received triple TIV vaccination developed an HAI titer, whereas only 13% of mice that received TIV once had an HAI titer above the detection limit. Influenza virus nucleoprotein (NP) is highly conserved between influenza virus strains and antibodies to NP have been suggested to contribute to heterosubtypic immunity (18–20). TIV used in our study was a split virus vaccine and through Western blot the presence of NP in the TIV was confirmed (Figure 1F). Therefore, we looked at induction of antibodies toward recombinant NP (Figure 1D). ELISAs were done with NP from the A/PR/8/34 laboratory virus strain, which is the same type of NP included in the TIV backbone. Again, we saw that 3x TIV resulted in the highest total IgG titer whereas a single TIV administration did not result in NP-specific IgG levels above background. Since the TIV also had a H3N2 component, we also looked at ELISA titers against H3 hemagglutinin from the HK68 strain (A/Hong Kong/1/1968), which is the HA of the 2nd challenge virus strain but was different from the vaccine component (Figure 1E). A single administration of TIV did not result in HK68 H3 HA-specific IgG titers. However, 3x TIV resulted in detectable total IgG levels that were higher than the control PBS group. This suggests that multiple vaccinations with TIV allowed mice to induce antibodies that cross-reacted with a different H3 HA from a virus strain that circulated multiple decades before.

### Single TIV Vaccination Is Infection-Permissive but Protects From Overt Morbidity and Inflammation During Experimental Influenza Virus Infection

**Figure 2:**
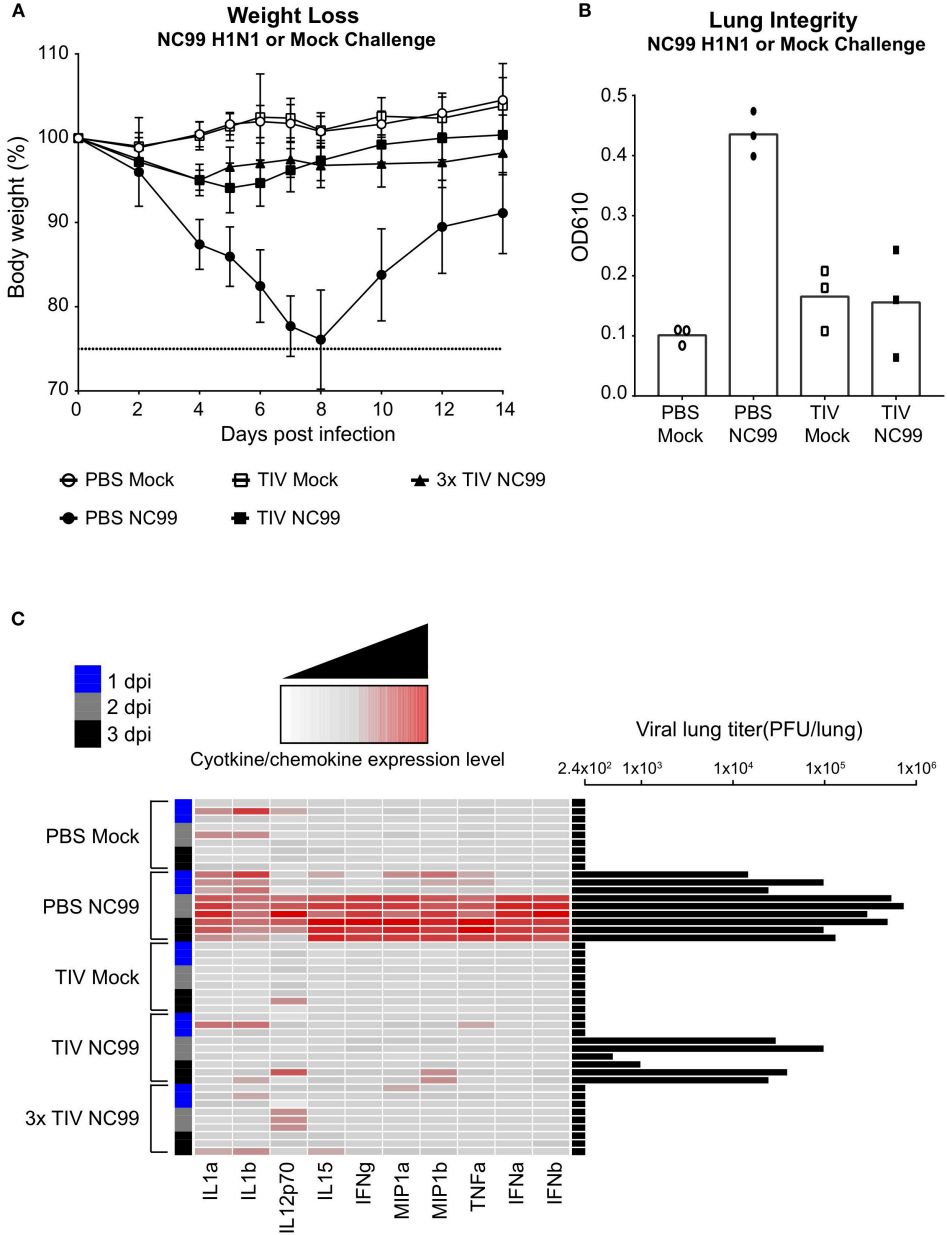
TIV vaccination provides protection against morbidity and inflammation caused by influenza virus infection. **(A)** Mice were given a homologous H1N1 virus infection or egg allantoic fluid as mock infection. The body weights of mice were recorded for 14 days after challenge. Animals that lost more than 25% body weight (dotted line) were euthanized for humane reasons. Symbols and error bars represent mean ± SD. **(B)** Lung integrity at 6 days post infection was observed using Evan's Blue Dye. Supernatant from BALF were measured at OD 610 nm. Each symbol represents one mouse. *n* = 3 mice/group. Bars represent mean. **(C)** Lung homogenates were prepared at day 1 (blue), 2 (gray), and 3 (black) post-challenge. Cytokine and chemokine expression levels were determined using a luminex cytokine bead array. Each column is a single cytokine/chemokine as indicated at the bottom of the heat map. Each row represents an individual mouse with vaccine and challenge status indicated on the left-hand side of the heat map. Lung viral titers (PFU/lung) were also measured through plaque assay. Titers were plotted as black bars on the right-hand side of the heatmap. *n* = 3 mice/dpi; *n* = 9 mice/group.

Three weeks after the last vaccination, each group of mice was either challenged with a sublethal dose of NC99 H1N1 virus, which matched the H1N1 component of TIV, or allantoic fluid as a mock control. Body weights were monitored for 14 days (Figure 2A). Both groups that were mock infected (PBS Mock, TIV Mock) showed no signs of body weight loss. The most loss (average of 24% of initial body weight) was observed in the group that received PBS followed by a challenge with NC99 (PBS NC99). Both groups that received TIV—either 1x or 3x—showed protection from disease against sublethal NC99 infection as compared to PBS controls. Despite being vaccinated, we saw a drop in body weight of about 6% at 4 days post infection (dpi) in the TIV NC99 group and about 5% weight loss at the same dpi for 3x TIV NC99. This also illustrated that the presence of higher HAI titers in the 3x TIV group (Figure 1C) did not correlate with enhanced protection from morbidity compared to 1x TIV during NC99 H1N1 infection in our mouse model.

To further look at the protection provided by TIV vaccination, viral lung titers as well as virus-induced cytokines, and chemokines were quantified at day one, two, and three post infection (Figure 2C). As expected, groups that were mock infected did not show any viral titers in the lungs. PBS NC99, the group that showed the highest morbidity, had the highest lung viral titers and a clear pro-inflammatory cytokine and chemokine profile from 2dpi on. Levels of interleukine (IL) 1α and IL1β went up immediately after infection in PBS NC99 mice. In the PBS Mock control group, we do see minor increase in levels of IL1 in some mice, which may reflect some minor inflammation due to administrating allantoic fluid intranasally. Interestingly, mice that were vaccinated once (TIV NC99) had detectable viral titers at 2 and 3 dpi, indicating that immunity provided by single TIV administration was infection-permissive. Lung viral titers in TIV NC99 mice were reduced 10-fold compared to the PBS NC99 control group. Since no virus was detected at 1 dpi in TIV NC99 mice, the virus quantified at 2 and 3 dpi was the result of virus replication rather than detection of initial inoculum virus. Although virus replication was allowed in mice that received a single TIV vaccination to some extent, this did not result in upregulation of pro-inflammatory cytokines and chemokines at the measured time points post infection. On the other hand, the 3x TIV NC99 group fully prevented viral replication to occur in the lungs, resulting in sterilizing immunity. The absolute concentrations of cytokines and chemokines that were measured in lung homogenates are given in Supplementary Figure 1.

Loss of lung integrity during influenza infection has been described and can be monitored by measuring levels of Evans Blue-stained serum albumin in bronchoalveolar lavage fluid (BALF) after intravenous injection as a proxy for increased lung permeability and edema. In contrast to TIV vaccinated animals, control PBS mice had enhanced levels of Evans Blue dye in BALF at 6 days post NC99 H1N1 infection (Figure 2B). Despite detectable virus replication in the lungs, single TIV vaccination prevented loss of lung integrity after infection as enhanced levels of Evans Blue dye were not detected in BALF of TIV NC99 mice. This further illustrates that single dose TIV vaccination can protect from virus-induced inflammation and pathology without inducing sterilizing immunity.

**Figure 3:**
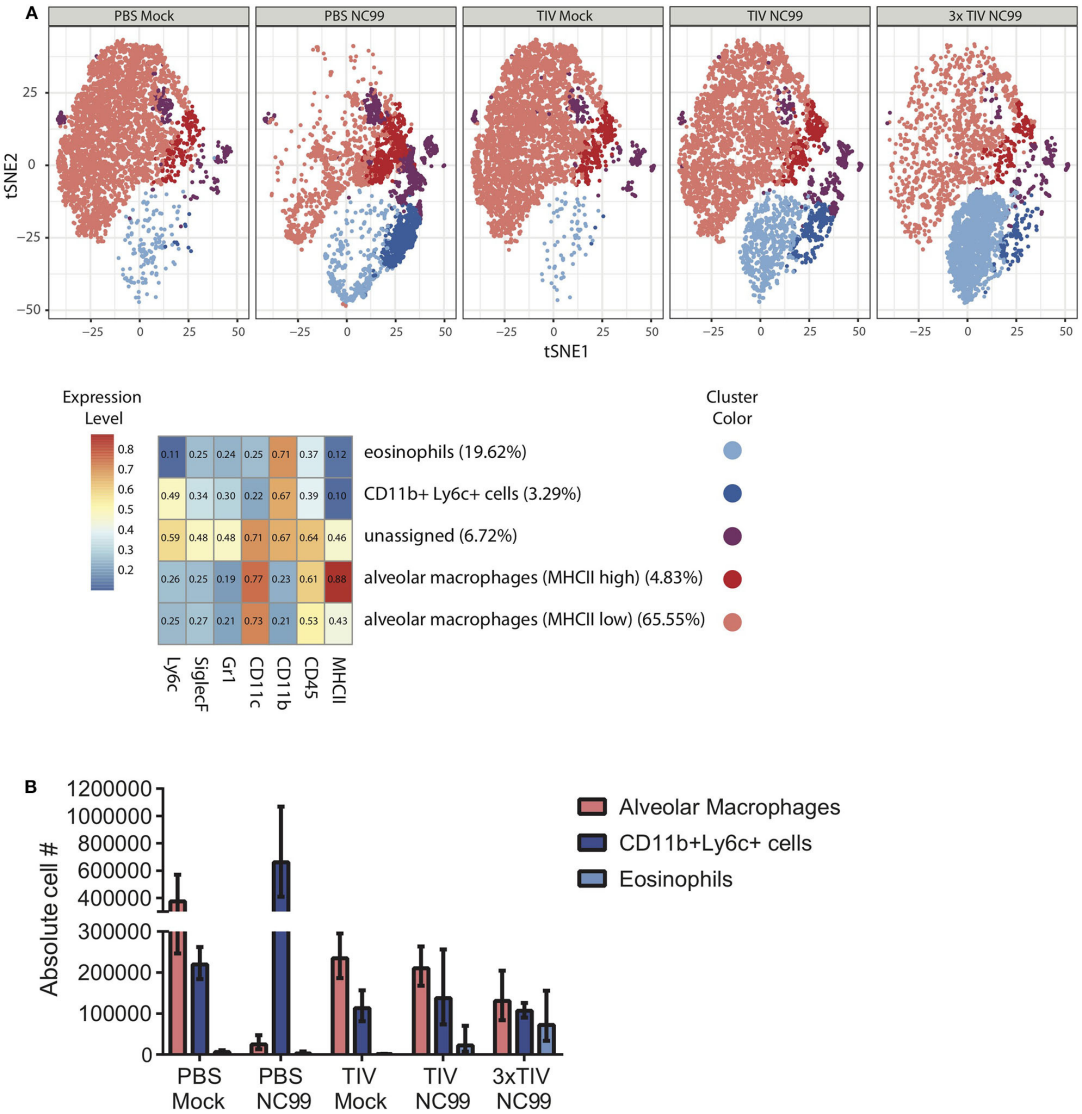
TIV vaccination affects the immune cell populations in the lung at 7 days post H1N1 virus infection. **(A)** Unsupervised analysis of immune cell populations was performed. Single cell suspensions from mouse lungs were stained for viability and surface markers. Live, SiglecF+ single cells were pregated to define immune cell populations by clustering cells based on their mean fluorescence intensities for different surface markers. Surface markers that were used are mentioned at the bottom of the heatmap. Z-scored mean fluorescence intensities are given from the respective clusters in the heatmap. Every row represents the surface expression profile for one cluster. Relative abundance of different clusters is given between parentheses after the cluster names. t-SNE plots for the five experimental groups were then generated. Each cluster was given different colors. Every plot represents subsamples of 1,000 cells/mouse with *n* = 5 mice/group. **(B)** Absolute cell counts of alveolar macrophages, Ly6c+ monocytes, and eosinophils were also quantified through flow cytometry. Bars represent means ± SD. *n* = 5 mice/group.

At 7dpi, we explored the cellular composition of lungs of vaccinated and unvaccinated mice (Figure 3). Numbers of Ly6c+ monocytes (CD45+ CD11c– SiglecF– CD11b+ Ly6c+ cells) were enhanced in PBS NC99 mice. On the other hand, TIV NC99 group had similar numbers to mock-infected animals, confirming the absence of enhanced inflammation in TIV mice (Figure 3B). These flow cytometry data were obtained from single cell suspensions of whole lungs without prior perfusion to remove blood cells. This may explain why basal levels of Ly6c+ monocytes were already quite high at baseline (PBS Mock). Complete depletion of alveolar macrophages by 7 dpi has been described in the BALB/c mouse influenza infection model (21, 22). Absolute numbers of alveolar macrophages (CD45+ CD11c+ SiglecF+ CD11b– cells) dropped drastically by 7 dpi in PBS NC99 mice (Figure 3B) while TIV NC99 animals were close to those of mock-infected animals. We also observed the recruitment of CD45+ CD11b+ CD11c– SiglecF+ cells after NC99 infection into the lungs of vaccinated mice, but not in PBS control mice. These surface markers suggest that this recruited cell population consists of eosinophils and this phenomenon is dose-dependent since 3x TIV results in more eosinophils than 1x TIV (Figures 3A,B). However, we cannot exclude that these higher levels of eosinophils are in the blood or the lung parenchyma/alveolar space since perfusion or intravascular staining was not done. Overall, vaccination with TIV prevented morbidity and inflammation caused by NC99 H1N1 virus infection. Moreover, vaccination followed by infection resulted in the recruitment of cells with the surface markers of eosinophils, even in the case of 3x TIV vaccination induced sterilizing immunity.

### TIV Vaccination Can Prime NP-Specific CD8+ T Cell Responses but Interferes With Virus-Induced CD8+ T Cell Responses

**Figure 4:**
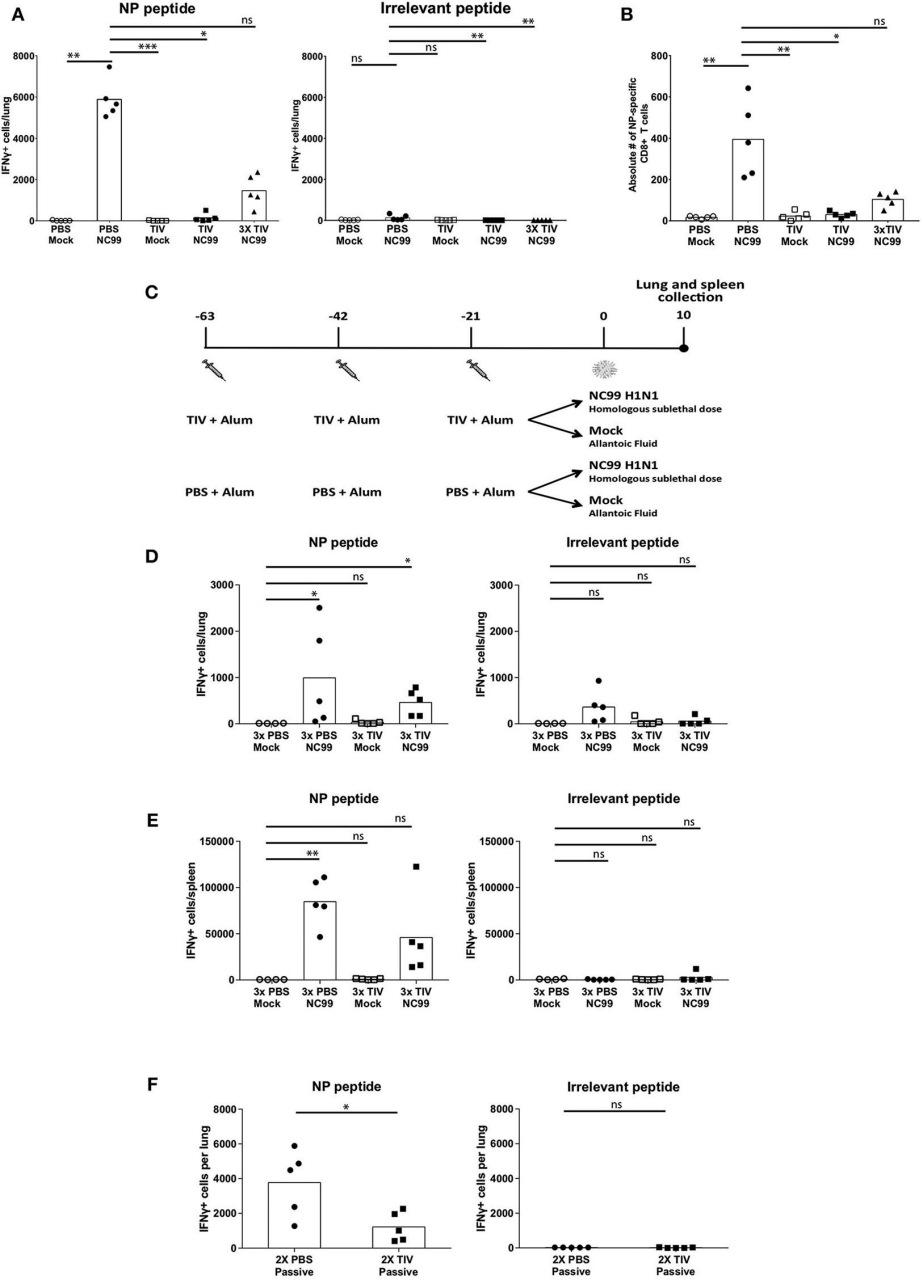
Cellular host immune responses are affected by pre-existing immunity given by vaccination and infection. **(A)** Lungs were collected to quantify antigen-specific T cells at 10 dpi. The cells isolated from the lungs were stimulated with either NP-derived peptide, TRYQTRALV (NP-specific), or irrelevant peptide. Each symbol represents the number if IFNγ-producing cells measure by ELISpot per mouse. Bars represent means. *n* = 5 mice/group. **(B)** NP-specific CD8+ T cells in the lungs were quantified by NP-specific pentamer staining. Each symbol represents counts per mouse. *n* = 5 mice/group. Bars represent mean. **(C)** Study design to look at the effect of repeat vaccinations with TIV. Mice were vaccinated three times with TIV or PBS with 3-week intervals. Each vaccination was adjuvanted with Alum. Three weeks after the last vaccination, both groups were challenged with either H1N1 NC99 or allanotic fluid. At day 10, both lungs and spleen were harvested for assessment of antigen specific T cell responses. **(D,E)** Cells isolated for lungs **(D)** and spleens **(E)** were restimulated with either NP-specific peptide or an irrelevant peptide. Individual symbols represent number of IFNγ+ cells/organ from one mouse. Bars represent mean. **(F)** After passive transfer of sera into naïve mice and challenged with NC99, lungs were collected and restimulated with NP-specific peptide or an irrelevant peptide for control. Each symbol represents a mouse. Bars represent means. *p < 0.05, **p < 0.01, ***p < 0.001, ns = not significant.

CD8+ T cells contribute to protection against viral infections by killing infected cells that present viral antigen in the context of MHC-I molecules. Influenza virus NP produced in the cytoplasm of infected cells is known to be conserved between different influenza subtypes (23). T cell recognition of this type of viral antigens can result in protective heterosubtypic immunity. In an ELISpot assay, we used an H2d-restricted NP-specific peptide (TYQRTRALV) and an irrelevant H2d-restricted peptide as a negative control to restimulate IFNγ secreting cells in Percoll-enriched single cell suspensions made from lungs or spleens at 10 dpi. Antigen derived from residual virus in lung samples from PBS NC99 mice can explain the low back ground seen in the ELISpot assay when restimulated with irrelevant peptide (Figures 4A,D). When comparing the two PBS vaccinated control groups (PBS mock vs. PBS NC99), mice infected with NC99 H1N1 mice had the most IFNγ+ cells (Figure 4A). Animals that received single TIV vaccination without NC99 challenge did not show detectable T cell responses at this time point. Despite the presence of replicating virus, numbers of IFNγ-secreting cells were close to background in TIV NC99 mice, suggesting that single TIV vaccination interfered with the induction of NP-specific T cell responses after infection. However, mice that received 3x TIV vaccinations had a mean value of 1,464 IFNγ+ cells in the lungs after NC99 challenge. Interestingly, we did not detect replicating virus in this experimental group shortly after infection (Figure 2C). This suggested that multiple administrations of TIV could prime for NP-specific CD8+ T cell responses in the periphery resulting in higher T cell responses in the lungs of 3x TIV NC99 mice.

To confirm that the IFNγ+ spots observed in the ELISpot assay were due to NP-specific CD8+ T cells, we also performed flow cytometry on a small fraction of the single cell lung suspension using TYQRTRALV-specific pentamers. When measured by flow cytometry, levels of NP-specific CD8+ T cells for the different experimental groups confirmed the results of the ELISpot assay (Figure 4B). To investigate if multiple vaccinations with TIV can result in detectable NP-specific IFNγ+ CD8+ T cells without the need of a virus infection, we performed an independent repeat experiment that also included a mock challenge control for 3xTIV mice (Figure 4C). Although we observed more experimental variation in this experiment, it was clear that the number of IFNγ+ spots in 3xTIV Mock mice was similar to back ground levels while in infected mice (3x TIV NC99) IFNγ + spots were detected. Thus, 3x TIV animals still needed an NC99 H1N1 infection in order to have detectable NP-specific CD8+ T cell responses in both lungs and spleen (Figures 4D,E).

It has been suggested by Kim et al. that antibodies induced by TIV vaccination can contribute to the induction of vaccine-specific CD8+ T cell responses (24). To investigate this in our BALB/c TIV vaccination/influenza challenge model, we passively immunized naïve animals via the intraperitoneal route with serum from BALB/c mice that received TIV twice. Twenty-four hours after serum transfer, mice were intranasally challenged with NC99 H1N1 virus and TYQRTRALV-specific CD8+ T cell responses were assessed by ELISpot assay at 10 dpi. Contrary to the report of Kim et al., passive transfer of TIV serum resulted in a reduction of pulmonary NP-specific T cell responses (Figure 4F). In conclusion, in this TIV vaccination/influenza challenge model, a single TIV vaccination or a passive serum transfer of TIV serum resulted in a reduction of virus-induced CD8+ T cell responses. Repeated TIV vaccination primed for CD8+ T cell responses, though to a lower extent than replicating virus, and still needed a nasal virus challenge to be detectable by ELISpot.

### Humoral Immune Responses Are Boosted by Influenza Virus Infection

**Figure 5:**
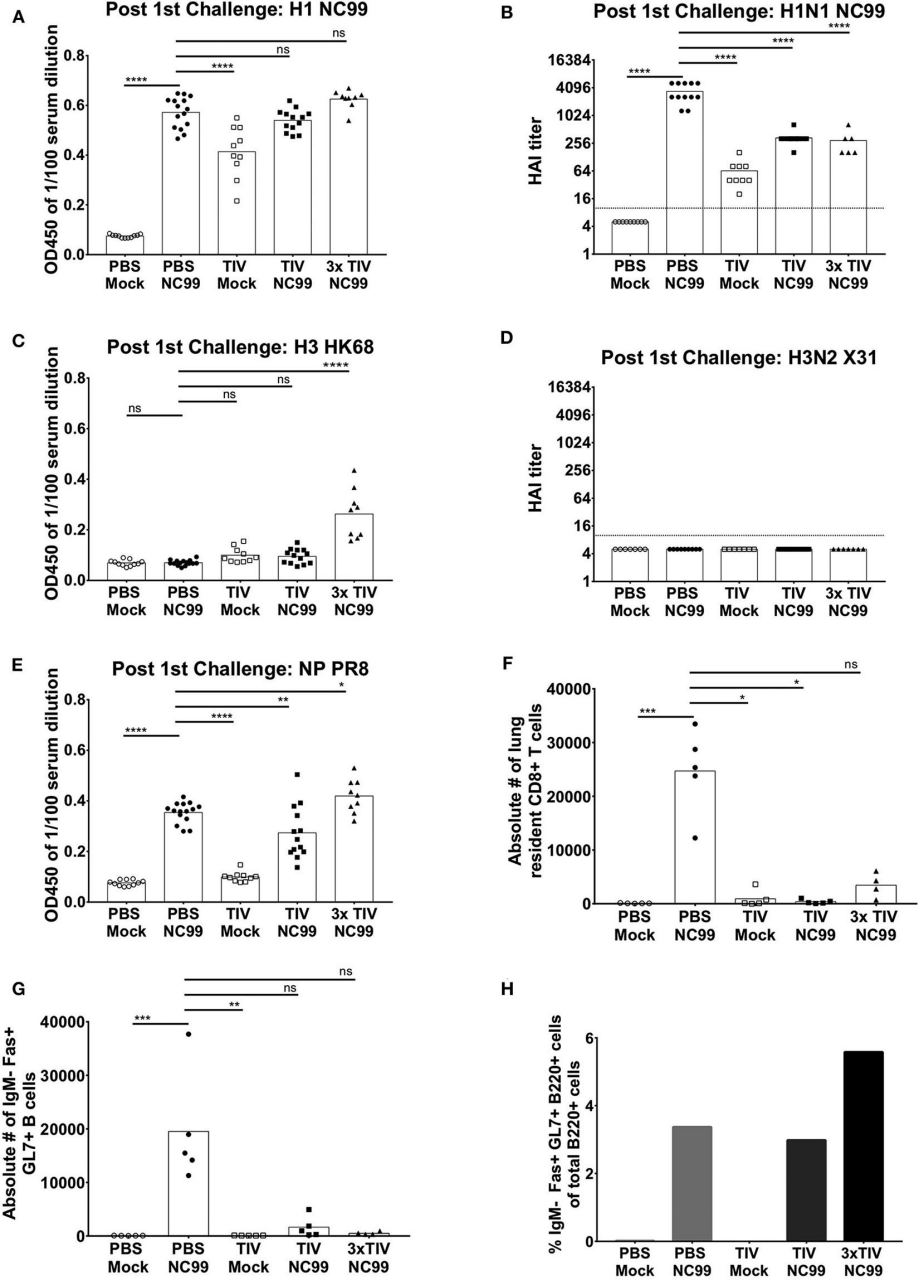
Humoral immune responses were boosted after NC99 virus challenge and TIV vaccination prevented the formation of GC B cells and TRM in the lungs after infection. **(A,C,E)** On day 26 serum were collected from individual mice and diluted 1/100 to perform ELISAs. Total IgG antibody titers against full length H1 NC99, H3 HK68 Has, and NP PR8 were analyzed. **(B,D)** HAI titers against H1N1 NC99 and H3N2 X31 viruses were also measured. Dotted line represents the limit of detection in the HAI assay. **(F)** Lung resident CD8+ T cells (CD3+, CD8+, CD44+, CD69+, CD103+ cells) were analyzed from lungs collected about 4 weeks after the first challenge (Day 27). Circulating immune cells were excluded by i.v. CD45 labeling. *n* = 5 mice/group. **(G)** Germinal center B cells are a hallmark of iBALT formation. Thus, lungs were harvested on Day 24 and CD3–, IgM–, B220+, Fas+, GL7+ cells were quantified through flow cytometry. *n* = 5 mice/group. **(H)** Germinal center B cells were also analyzed in lymph nodes. From the same mice used to harvest lungs, mediastinal lymph nodes were collected and pooled per group. Pooled samples were analyzed by flow cytometry and percentages of GC B cells were plotted. All bars represent means and symbols represent an individual mouse. *p < 0.05, **p < 0.01, ***p < 0.001, ****p < 0.0001, ns = not significant.

A single TIV vaccination is infection-permissive, but blunts cellular host immune responses after influenza infection. Therefore, we wanted to test to what extent the humoral host immune response after influenza infection is affected by TIV vaccination. Twenty six days after the initial NC99 H1N1 challenge, sera from each group were collected to measure humoral responses through ELISA and HAI. ELISAs were performed with NC99 H1 HA that matches the H1N1 strain in the TIV vaccine and the NC99 challenge virus. All experimental groups that were given NC99 H1N1 challenge virus had high H1-specific total IgG titers, including the control animals that received PBS instead of TIV (Figure 5A). The latter group even had the highest HAI titers at this time point (Figure 5B). This suggests that experiencing an active NC99 H1N1 infection induces H1-specific IgG antibodies to an equal extent, or even better, titer in naïve animals compared to animals that were TIV primed. H1-specific ELISA titers were on average slightly higher for 3x TIV NC99 mice compared to TIV NC99 mice. Although antibody levels were similar to those that received challenge without TIV, HAI titers were not higher in animals that were either given TIV once or three times (Figure 5B). Interestingly, at this time point (5 weeks post vaccination), a single administration of TIV without NC99 challenge resulted in detectable H1-specific ELISA and NC99-specific HAI titers in all animals tested. This was not the case when the same assays were performed 2 weeks post vaccination (Figures 1B,C) and illustrates that humoral immune responses following TIV vaccination continue to build up and do not reach a maximum at 2 weeks post vaccination.

Before the second challenge, we also measured antibody responses against HK68 H3 hemagglutinin and HAI titers against X-31 virus that carries the HK68 H3 HA. Sera from both PBS Mock and PBS NC99 mice showed no antibody binding toward HK68 H3 HA (Figure 5C). The H3N2 strain in the TIV vaccination (A/New York/55/2004) does not match the X-31 H3N2 strain and we observed very low anti-H3 HK68 binding after one vaccination with TIV, irrespective of a NC99 H1N1 challenge. Animals that were vaccinated three times with TIV, mounted higher levels of anti-HK68 H3 antibodies and antibody levels did not increase further over time (Figures 5C, 1E). None of the experimental groups developed detectable HAI titers against X-31 H3N2 virus after the NC99 H1N1 challenge (Figure 5D).

NC99 H1N1 infection induced NP-specific antibodies (Figure 5E). Unlike the antibody levels against NC99 H1 HA, no enhancements of NP antibody levels were monitored over time for the TIV Mock group. NC99 H1N1 infection boosted the level of anti-NP antibodies in TIV-vaccinated animals, with 3xTIV mice having the highest levels of NP-specific IgG antibodies. This was in line with the observation in Figure 1D where multiple TIV administrations already resulted in detectable NP antibody titers.

### TIV Vaccination Interferes With the Establishment of Infection-Induced Pulmonary Mucosal Germinal Center B Cells and TRM but Not Germinal Center B Cells in Peribronchial Lymph Nodes

The presence of inducible bronchus associated lymphoid tissue (iBALT) and tissue resident memory (TRM) T cells have been shown to correlate with heterosubtypic immunity (15, 17, 25–27). Therefore, 1 week before the heterosubtypic challenge, lungs and lymph nodes were harvested to analyze the presence of these cell populations. First, we focused on germinal center (GC) B cells (IgM– Fas+ GL7+ B220+ cells), which are a hallmark of iBALT. Lungs were harvested, taking care not to co-isolate peribronchial lymph nodes, 24 days after challenge and processed to single cell suspensions. After mice were challenged with NC99 H1N1 virus, PBS vaccinated mice showed an induction of pulmonary B cells (Figure 5G). In contrast, mice vaccinated with TIV once or three times had almost no GC B cell levels above back ground after NC99. The absence of GC B cells in lungs after infection in TIV mice, however, was not reflected in pooled peribronchial lymph nodes. Percentages of GC B cells were equally high for single TIV vaccinated animals after challenge, and were even higher in 3x TIV mice (Figure 5H).

Next, we focused on tissue-resident CD8+ T cells (TRM; CD3+ CD8+ CD44+ CD103+ CD69+ cells) in lungs after exclusion of circulating T cells by i.v. labeling with CD45 antibody. Similar to the levels of GC B cells, we also saw high levels of lung-resident CD8+ T cells in lungs of PBS NC99 mice at 27 dpi (Figure 5F). Similar to our observation of CD8+ T cells at 10 dpi (Figures 4A,B), TIV vaccination interfered with the induction of lung resident CD8+ T cell responses after virus infection.

### Virus-Induced Host Responses Correlate With Heterosubtypic Immunity During Re-Challenge

**Figure 6:**
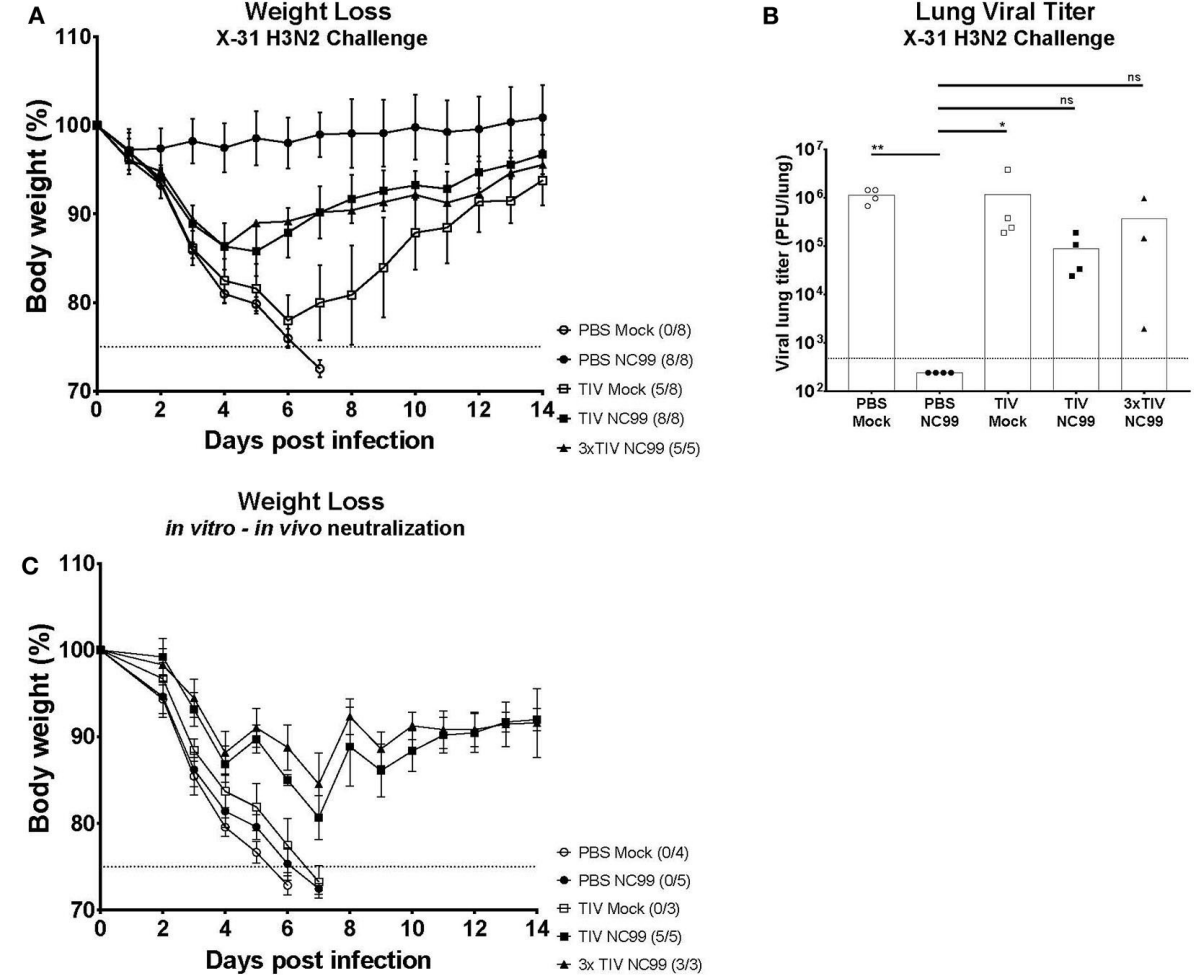
Protection against lethal X31 H3N2 virus challenge is observed in mice with pre-existing immunity provided by vaccination and infection. **(A)** 4 weeks after the first challenge (Day 28), all groups received a second challenge with a lethal dose of X-31 H3N2. The body weighs of mice were monitored for 14 days. Animals that lost more than 25% body weight (dotted line) were euthanized for humane reasons. Each symbol represents the mean body weight per group. Error bars represent SD. The number of mice that died over total number of mice per group is shown next to each symbol. **(B)** 5 days post X-31 challenge (Day 35), lungs were collected to quantify viral titers. Individual symbol is an individual mouse, bars represent means and dotted line represents the limit of detection (480 PFU/lung). **(C)** Body weights from each group were monitored for 14 days after *in vitro-in vivo* neutralization assay was performed. Naïve mice received the same lethal dose of H3N2 X-31 virus after *in vitro* incubation of virus with different groups of pooled sera for 1 h at 37°C. Each symbol represents the mean body weight of naïve mice that received pooled sera from specific groups. Animals that lost more than 25% body weight (dotted line) were euthanized for humane reasons. The number of mice that died over total number of mice per group is shown next to each group name. *p < 0.05, **p < 0.01, ns = not significant.

Influenza virus infection results in the induction of strong cross-reactive immune responses that correlate with heterosubtypic immunity in mice (6, 26). To assess the effect of pre-existing immunity on virus-induced heterosubtypic immunity, all experimental groups were challenged with 5LD_50_ of X-31 H3N2 virus (Figure 1A). All mice in the control group (PBS Mock) succumbed to virus infection by day 7 post challenge with X31 H3N2 virus (Figure 6A). These animals also had high mean lung viral titers (9.6 × 10^5^ PFU/lung) on 5 days post X-31 infection (Figure 6B). In contrast, animals that were not vaccinated with TIV and showed high morbidity after NC99 H1N1 challenge (PBS NC99) were the group that were best protected from morbidity and mortality after X-31 H3N2 re-challenge (Figure 6A). PBS NC99 mice also controlled viral replication in the lung completely (Figure 6B). The TIV Mock group had high viral titers by day 5 post re-infection (Figure 6B), lost an average of 22% of their body weight by day 6, and overall lost 3 out of 8 mice (Figure 6A). Compared to TIV Mock, protection against X-31 H3N2 re-infection was enhanced in TIV vaccinated mice when combined with a sublethal NC99 H1N1 challenge. Although both TIV NC99 and 3x TIV NC99 groups lost about 14% body weight before recovering, all mice survived the lethal dose of X-31 H3N2 virus (Figure 6A). Both groups also had viral replication in the lungs but at slightly reduced titers than mice that had not experienced the NC99 H1N1 infection before (TIV Mock and PBS Mock) (Figure 6B). Interestingly, no additional benefit in terms of reduction of virus titers or morbidity was seen for mice that received TIV three times compared to mice that received TIV only once.

### Correlates for Heterosubtypic Immunity Are Skewed From Cellular to Humoral Immune Factors by Pre-existing Immunity

Both TIV NC99 and 3x TIV NC99 lacked pulmonary GC B cells and TRM. Nonetheless, these mice were fully protected from mortality after rechallenging them with a lethal dose of X-31 H3N2 virus. Since TIV NC99 and 3x TIV NC99 mice do not have lung resident GC B cells and TRM, we wanted to investigate if heterosubtypic immunity was covered by the humoral branch of the immune system. Hereto we performed an *in vitro*-*in vivo* neutralization assay in which we infected naïve mice with a mixture of X-31 H3N2 virus (5LD50) pre-incubated with sera from the different experimental groups. Mice that received sera from PBS Mock, the group that showed no humoral or cellular immunity, all succumbed to death by day 6 (Figure 6C). Interestingly, animals that received PBS NC99 sera, the group that showed the best protection against X-31 H3N2 rechallenge, also succumbed to death by day 7. TIV Mock sera were also not protective after X-31 virus challenge. Serum from TIV NC99 and 3x TIV NC99, however, was protective against X-31 H3N2 virus with complete survival and reduced morbidity. Similar to what we observed for protection in actively vaccinated mice (Figure 6A), sera from 3x TIV mice did not provide superior protection in the *in vitro*-*in vivo* neutralization assay compared to sera from 1x TIV mice. In summary, these data suggest that H1N1 challenged TIV vaccinated animals rely more on cross-reactive protective serum antibodies than cellular immunity for survival during the lethal X-31 H3N2 re-infections.

## Discussion

Recent research suggests that host immune responses in mice that have experienced multiple immunological stimuli before may differ from those observed in naïve mice (28). Pre-existing immunity toward influenza viruses is present in the human population due to repeated exposure to influenza virus antigens through infection and vaccination. In this work, we investigated the effect of pre-existing immunity on virus-host immune responses during H1N1 influenza A virus infection, and how this correlates with protection during re-infection with an influenza virus of a different subtype. Pre-existing immunity was provided by TIV vaccination that was titrated to provide protection in the absence of complete virus neutralization, as was evident from the replicating virus observed in the lungs of TIV NC99 mice. A single TIV vaccination, but not 3x TIV, was inefficient to induce strong antiviral antibody titers against HA or NP (Figures 1B–D). Antibody titers against H1 NC99 HA were induced (PBS NC99) or further boosted (TIV NC99) after infection. In our study, only total IgG levels were measured through ELISA. Other antibody isotypes and subtypes have been shown to play different roles in neutralizing influenza viruses (29). Further characterization to see if a certain antibody iso/subtype, such as IgG2a and IgM, correlates best with protection would be interesting to analyze in the future for the 3x TIV group post-vaccination and all groups after NC99 H1N1 infection. Despite the presence of NP antigen in the vaccine (Figure 1F), mice did not mount detectable NP-specific CD8+ T cell responses in the periphery (spleen) or lungs (Figures 4D,E) upon 1x TIV or 3x TIV vaccinations if not followed by an active infection. However, if vaccination is followed by virus challenge, 3 vaccinations with TIV primed for NP-specific T cells, as evidenced from the higher recall response at 10 dpi with NC99 H1N1 (Figures 4A,B,D,E). These T cell responses were still lower compared to mice that were not given TIV and got very sick after NC99 H1N1 infection. This means that repeated TIV vaccination can prime for NP-specific T cell responses, but can also interfere with the induction of NP-specific T cell responses by preventing or limiting virus replication. Since infection is needed to mount detectable CD8+ T cell responses, viral antigen encounter in the lungs seems to be important. It has been suggested that induction of influenza virus-specific CD8+ T cells by infection can be facilitated by uptake of antibody-antigen immune complexes (24). However, in our passive transfer and challenge model, transfer of TIV-immune serum resulted in lower numbers of pulmonary NP-specific CD8+ T cells (Figure 4F). Similar to active vaccination, the serum that was transferred to naïve mice likely restricted virus replication and limited the amount of viral antigen. While 1x TIV did not provide sterile immunity, it is intriguing that high levels of CD8+ T cells were not induced after virus challenge. It has been shown that CD103+ dendritic cells (DC) are critical for CD8+ T cell priming in the mouse model (30). Animals that were in the TIV NC99 group did not have high levels of cytokines in their lungs. Thus, due to the lack of favorable cytokine/chemokine milieu after infection in TIV vaccinated animals, it is possible that absence of CD8+ T cell responses is a result of inefficient DC activation. Further studies are needed to tease out if and how priming of CD8+ T cell responses is being impaired in these mice, and if there is an eventual role for regulatory T cells in this.

Interestingly, mice that received TIV three times still showed minor body weight loss on average after infection, which was comparable to mice that received TIV only once (Figure 2A). On the other hand, mice that lacked HAI titers (1x TIV) were equally protected from body weight loss as mice that showed serum HAI titers (3x TIV). Therefore, in this experiment, HAI titer was not a reliable correlate of protection against a homologous virus infection. We hypothesize that immune mechanisms other than neutralizing antibodies like innate immune responses immediately after infection and non-neutralizing antibodies may be responsible for the absence of inferior protection for the 1x TIV group compared to the 3x TIV group. On the other hand, both 1x TIV and 3x TIV mice had low cytokine and chemokine levels during the first days post infection similar to mock-infected animals (Figure 2C).

Additionally, we observed that after infection with NC99, the composition of myeloid populations in the lung was skewed in TIV vaccinated mice. TIV-mediated protection from morbidity was associated with background levels of CD11b+Ly6c+ monocytes (Figure 3), whereas this population was enriched in PBS NC99 mice. This illustrates again that despite the absence of high antiviral serum titers or detectable T cell responses, a single TIV vaccination was protective and prevented strong inflammatory responses. Alveolar macrophages are a first line of defense during influenza virus infection. In the BALB/c model, experimental infection has been shown to result in alveolar macrophage cell death (22, 31). Despite being infection-permissive, TIV vaccination prevented complete loss of alveolar macrophages and preserved lung integrity by 7 dpi (Figure 3). We have recently published the observation that infection in TIV-vaccinated mice resulted in pulmonary recruitment of CD45+ SiglecF+ CD11b+ CD11c– cells, which is a typical surface marker combination for eosinophils (32). In this study, we confirmed this phenomenon as eosinophils were present in the lungs of mice that received TIV vaccination followed by NC99 infection, but not in unvaccinated mice (Figure 3). We also observed an antigen dose-dependency since eosinophil levels were higher when mice were vaccinated multiple times before infection (3x TIV NC99). The animals used in our study were BALB/c mice that are prone to type 2-skewed responses. Thus, it is possible that the increase in eosinophil levels we observe are BALB/c specific. However, in a similar vaccination-challenge model used in C57BL/6 mice, which are more type 1-skewed, resulted in similar increase of eosinophils in the lungs of mice that were vaccinated and infected (data not shown). Based on our result, pulmonary eosinophil recruitment correlated with experimental groups that were protected by vaccination. The recruitment of eosinophils to the lungs upon infection in vaccinees has a negative connotation in the context of respiratory viruses. This is in part because of the negative outcome of the respiratory syncytial virus vaccine trial which was associated with eosinophilia after infection in vaccinated children (8). However, recent research suggests that eosinophils can also have antiviral properties, directly through engulfment of virus or indirectly through local antigen presentation to T cells (33, 34). Thus, the increase of eosinophils in the lungs may play a role in virus control or disease inhibition. Further research on the recruitment and functionality of eosinophils against influenza virus are ongoing. We are currently investigating if alveolar macrophages, saved from cell death after vaccination, can function as a chemokine source during infection for recruitment of eosinophils.

Previous studies have shown that pulmonary infections can result in inducible bronchus associated lymphoid tissue (iBALT) in mice (15). The formation of tertiary lymphoid organ is characterized by the presence of class-switched germinal center B cells and *de novo* production of antibodies. Presence of iBALT has also been shown to correlate with HSI in the mouse model (17). In our study, TIV vaccination prevented the formation of iBALT as levels of GC B cells were highly reduced or absent in lungs of TIV mice after NC99 H1N1 infection (Figure 5G). This was in line with previous reports that vaccination can interfere with iBALT formation (26). When peribronchial lymph nodes where harvested for GC B cell quantification, the negative effect of TIV vaccination on iBALT formation was not reflected in the percentage of GC B cells (Figure 5H). Based on our results, TIV vaccination could either prime GC B cells, which are then recruited to the peribronchial lymph nodes upon infection, or vaccination does not interfere with induction of GC B cells upon influenza virus infection in the peribronchial lymph nodes. Along with iBALT, cross-reactive CD8+ T cell responses have been shown to contribute to HSI (35). CD8+ T cells that target conserved influenza antigens, like the nucleoprotein, can provide protection in both humans and animal influenza models (5, 36–40). During natural influenza virus infection, CD8+ T cell responses are induced and can obtain a lung resident memory phenotype. These tissue resident memory T cells can act as the first line of adaptive immune defense during reinfection. Previous studies have shown that vaccination may delay or prevent the onset of such T cell responses by preventing the host to experience a productive influenza virus infection (10, 26, 41). As seen previously with the iBALT formation in the TIV NC99 mice, establishment of tissue resident CD8+ T cells was also prevented in TIV vaccinated mice, despite the presence of replicating virus, and thus viral antigen, in the lungs (Figure 5F). A possible explanation can be the lack of a pro-inflammatory cytokine and chemokine milieu in the lungs of vaccinated mice (Figure 2C). IL-1 signaling early in infection has been shown to be crucial for induction of iBALT (16). IL-1 also plays a role in licensing pre-committed memory T cells to produce effector cytokines and helping in the establishment of TRM in lungs after adenoviral priming (25, 42). In our study, early IL1 signaling during the first 3 days post NC99 infection was observed in non-vaccinated animals (Figure 2C). On the other hand, IL1 production was mainly absent in mice vaccinated with TIV after infection. This correlated with the presence and absence of pulmonary GC B cells and TRM in PBS NC99 and TIV NC99, respectively (Figures 5F,G). Furthermore, cytokines such as IL12 and IL15 are known to be involved in CD8+ T cell activation and in the induction of T cell memory responses (43, 44). Enhanced levels of IL12 and IL15 were observed after infection in PBS NC99 mice, which is the group that had high levels of TRM in the lungs (Figure 2C). Finally, Ly6c+ monocytes can play an important role in antigen uptake and presentation to T cells in the lung and interaction between pulmonary monocytes and effector T cells in the lung are important for establishment of TRMs (45). TIV vaccination prevented Ly6c+ monocyte levels to rise above background levels upon NC99 infection (Figure 3). Only PBS NC99 mice, not TIV NC99 or 3x TIV NC99 mice, had enhanced levels of Ly6c+ monocytes at 7 days post infection which correlated with the presence of pulmonary TRM 4 weeks post NC99 challenge.

Presence of pulmonary GC B cells and TRM in mice that showed strong morbidity after H1N1 infection (PBS NC99) correlated with optimal protection during re-infection with a lethal dose of X31 H3N2 virus. Although TIV vaccination prevented the buildup pulmonary GC B cells and TRM after NC99 challenge, better protection from mortality after the second challenge with X31 H3N2 virus was observed in TIV NC99 and 3x TIV NC99 compared to TIV Mock animals, be it with higher morbidity compared to PBS NC99 animals (Figure 6D). This suggests that HSI seen in these different groups may correlate with other immune factors than lung-resident B or T cell responses. These immune factors are serum-transferable and therefore most likely antibody-mediated. As expected, humoral responses were boosted upon NC99 H1N1 infection. Boosting of vaccine-induced immune responses by virus exposure can theoretically contribute to protection during reinfection if circulating viruses do not show too much antigenic drift or induced antibodies target conserved epitopes (46). Interestingly, from our *in vitro-in vivo* neutralization assay, a combination of TIV vaccination and H1N1 infection were needed to induce cross-reactive immune sera that could prevent mortality against a heterosubtypic X31 H3N2 influenza virus in naïve mice. This suggests that vaccination with TIV primes cross-reactive antibody responses that are further enhanced by infection. In this context, it would be very interesting to see if the correlate shift from cellular to cross-reactive humoral immune responses that we observed can be extended to other influenza vaccine types like live-attenuated influenza virus vaccines (LAIV). Protection provided by LAIV, which mimics best natural infection, does not always correlate well with humoral immunity but is suggested to rely also on the induction of cellular immune responses in this age group (47). A study done by Zens et al. has also shown that mice that were vaccinated with LAIV were able to induce TRMs that can also provide cross-reactive protection against influenza viruses (48). Pre-existing immunity in our study was induced by TIV, which is known to induce humoral immune responses but is a poor inducer of T cell responses.

In conclusion, we illustrated that pre-existing immunity modulated host immune responses during influenza virus infection. Importantly, presence of pre-existing immunity can shift correlates of HSI from cellular toward humoral immune mechanisms, like cross-reactive sera. This has important implications for universal vaccine design and universal vaccination strategies. Since the level of pre-existing immunity in the human population changes with age, it may be advisable that universal vaccine approaches are tuned more toward the target population and age group. The very young of age are left unprotected with no influenza virus-specific pre-existing immunity after maternal antibodies wane. For this population, cross-reactive tissue resident cellular immunity may provide better protection against influenza virus infections. Furthermore, this will contribute to broad protection against influenza viruses during the first years of life. The elderly population, on the other hand, have a broad and cross-reactive but infection-permissive antibody repertoire against new influenza viruses and induction of cross-reactive cellular immune responses in this age group is more challenging (8, 49). Therefore, expanding the cross-reactive humoral branch of the immune system may be more feasible in this age group.

## Data Availability Statement

The datasets generated for this study are available on request to the corresponding author.

## Ethics Statement

The animal study was reviewed and approved by Icahn School of Medicine at Mount Sinai Institutional Animal Care and Use Committee.

## Author Contributions

MS: conceptualization, design of the study, and supervision. AC, LI, and MS: methodology and formal analysis. AC and MS: investigation, manuscript preparation, and visualization. MS, SS, FK, and AG-S: resources. MS and AG-S: funding acquisition.

## Conflict of Interest

The Icahn School of Medicine at Mount Sinai owns patents in the field of influenza virus vaccines on which AG-S and FK are inventors. The remaining authors declare that the research was conducted in the absence of any commercial or financial relationships that could be construed as a potential conflict of interest.

## Supplementary Material

Supplementary Figure 1 Concentrations of chemokine and cytokines measured after sublethal H1N1 NC99 infection. Cytokine and chemokine expression levels were determined on lung supernatant using a luminex-based 11-plex cytokine bead array or by classical ELISA (IFNa and IFNb). Concentrations for each cytokine/chemokines were plotted for each group after 1, 2, and 3 days post 1st challenge as indicated in Figure 1A. Horizontal lines represent means. Each symbol represents an individual mouse.
