# Supplementary figures and images for: Non-sterilizing, Infection-Permissive Vaccination With Inactivated Influenza Virus Vaccine Reshapes Subsequent Virus Infection-Induced Protective Heterosubtypic Immunity From Cellular to Humoral Cross-Reactive Immune Responses
Source: Front Immunol. 2020 Jun 9;11:1166. doi: 10.3389/fimmu.2020.01166 (PMC7296151; doi:10.3389/fimmu.2020.01166)

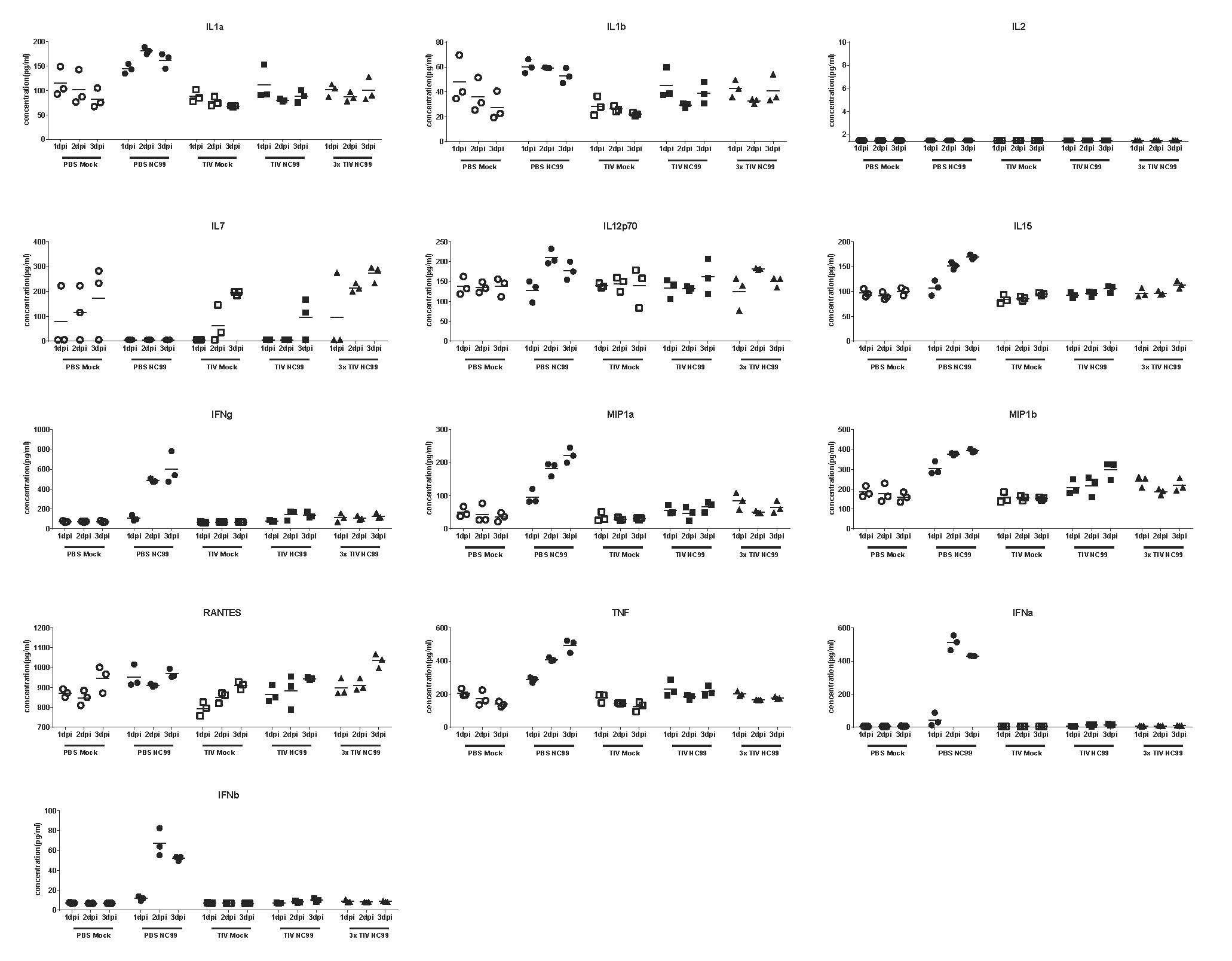

Supplement: Supplementary Figure 1 — Concentrations of chemokine and cytokines measured after sublethal H1N1 NC99 infection. Cytokine and chemokine expression levels were determined on lung supernatant using a luminex-based 11-plex cytokine bead array or by classical ELISA (IFNa and IFNb). Concentrations for each cytokine/chemokines were plotted for each group after 1, 2, and 3 days post 1st challenge as indicated in Figure 1A. Horizontal lines represent means. Each symbol represents an individual mouse. [file Image_1.JPEG]
